# Part 2: A Sector-Wide Survey of UK/British Isles Shelter Organisations Caring for Cats: Caregiver-Reported Approaches to Assessments, Behaviour Management and Homing Decisions

**DOI:** 10.3390/vetsci13060590

**Published:** 2026-06-18

**Authors:** Lauren R. Finka, Ana M. Barcelos, James Waterman, Avni Bhatia, Jenni L. McDonald, Rae Foreman-Worsley, Beth Skillings

**Affiliations:** 1 Feline Welfare Research Team, Cats Protection, National Cat Centre, Haywards Heath RH17 7TT, UKjames.waterman@cats.org.uk (J.W.); avni.bhatia@cats.org.uk (A.B.); jennifer.mcdonald@cats.org.uk (J.L.M.); rae.foreman-worsley@cats.org.uk (R.F.-W.);; 2 RedPony Analytics, Caernarfon LL55 4EP, UK; 3 Birkbeck College, University of London, London WC1E 7HX, UK

**Keywords:** welfare, rescue, rehoming, domestic cat, Felis catus, unowned cats, stray cats, feral cats

## Abstract

Shelter organisations take responsibility for the care, assessment and homing of large numbers of domestic cats from diverse backgrounds. However, not all cats that come under shelter care are suited to close human-cohabitation or to certain types of human-domestic lifestyles. Shelter stakeholders may undertake decision-making processes to determine how each cat should be managed and where they should go next. These processes may lead to different cat welfare experiences and long-term outcomes depending on how they occur, yet little is known about current approaches. The aim of this study was to characterise current approaches to cat and adopter assessments, behaviour management and homing decisions across the British Isles shelter sector, considering reported practices against sector minimum standards where applicable. A total of 393 quantitative and qualitative responses from employees and volunteers were received. Responses indicated that overall, stakeholders were consistently undertaking cat and prospective adopter assessments, with subsequent information used to support cat management, decision-making and homing. However, the degree of standardisation and objectivity associated with these processes was unclear, with considerable variation in approaches, including certain practices potentially associated with poor cat welfare outcomes identified. Examples include exposure to stressful handling and behavioural interventions and assessment ‘tests’, prolonged stays for harder-to-home cats and potentially suboptimal homing decisions for cats not suited to domestic ‘pet’ lifestyles. Identified opportunities to support welfare-friendly processes at the individual level include more consistent use of cats’ within-shelter behavioural presentations as grounds for ‘pet’-suitability assessments, and careful and consistent application of cat-labelling and terminology. It is also recommended that care is taken to ensure methods of cat assessments, behavioural interventions and homing decisions are pragmatic and optimised to positive cat welfare outcomes.

## Simple Summary

Shelter organisations take responsibility for the care, assessment and homing of large numbers of domestic cats from diverse backgrounds. Not all cats that come under shelter care are suited to close human-cohabitation, and shelter stakeholders may undertake decision-making processes to determine how each cat should be managed and where they should go next. These processes may lead to different cat welfare experiences and long-term outcomes depending on how they occur, yet little is known about current approaches. The aim of this study was to characterise current approaches to cat and adopter assessments, behaviour management and homing decisions across the British Isles shelter sector. A total of 393 quantitative and qualitative responses from employees and volunteers were received. Responses indicated that overall, stakeholders were consistently undertaking cat and prospective adopter assessments, with subsequent information used to support cat management, decision-making and homing outcomes. However, the degree of standardisation and objectivity associated with these processes was unclear, with considerable variation identified, including certain practices potentially associated with poor cat welfare outcomes. Various opportunities to support welfare-friendly decision-making and outcomes at the individual level are identified.

## 1. Introduction

At a species level, domestic cats exist across a broad spectrum of human sociability and occupy diverse lifestyles involving varying degrees of human association and management [1,2,3]. Domestic cats are the most popular companion animal in the UK and typically live in close cohabitation with humans, where they may provide a range of complex social and emotional benefits to their caregivers [4]. However, there are also large populations of unowned free-living cats that may either have no or limited contact with and reliance on humans [5]. Many cats may also be considered ‘semi’-owned; receiving regular supplemental feeding from community members and engaging in social interactions, but essentially remaining free-living and with limited human–social expectations placed upon them [1,6]. The implications of this diversity at an individual level are that many cats are not suited to close cohabitation with humans and confined lifestyles, and some cats are suited to certain types of human-domestic lifestyles and not others [7,8,9].

Shelter organisations admit, provide care for, and house substantial numbers of cats that may reflect this species-level diversity within their populations [10,11,12]. With either limited or no background information on many admitted cats [13,14,15,16] and generally limited resources to support the number of cats for which help is requested [17,18,19], providing the necessary individualised care to these diverse populations within-shelter can be very challenging. Shelter environments are also associated with inherent welfare risks to cats, including acute and chronic stress and various infectious and non-infectious diseases [20,21,22]. These risks are exacerbated by inappropriate housing and husbandry conditions [23,24,25,26,27], length of stay [20,22,23,28] and cat individual differences, including previous lifestyles [13] and limited human sociability [10].

Applying general best practice approaches to cat care and shelter stress management at a population level is therefore important, but to meaningfully and effectively reduce welfare risks, this also needs to be combined with an understanding of each cat as an individual. A crucial component of reducing welfare risks also relies on shelter stakeholders selecting the appropriate management pathway for each cat that they take responsibility for, including Trap-Neuter-Return/Relocate (TNR/R), adoption and euthanasia. This is in addition to admitting cats into shelters selectively based on current capacity and each cat’s individual characteristics, as well as securing a suitable post-shelter environment for them (i.e., the location into which a cat is released, the type of people and domestic lifestyle a cat is homed to). The negative consequences of applying inappropriate management pathways and homing decision-making include poor shelter cat welfare, increased length of stay and ineffective resource allocation [24,29,30], poor cat quality of life and human–cat relationships [31,32,33,34], and potentially more cats returned to shelters [35,36].

Undertaking individual cat assessments that provide valid and reliable information to assist in appropriate cat management and decision-making is therefore a critical component of shelter cat welfare management. However, the large majority of the shelter sector within the British Isles is currently unregulated, and there is a lack of detailed guidelines and resources, accredited training courses and related professional roles to support the sector to operate within best practice cat welfare frameworks. The charitable Association of Dogs and Cats Homes (ADCH) (About The Association Of Cats And Dogs Homes (https://adch.org.uk/minimum-standards-2/, accessed on 16 May 2026)|Registered UK Charity (https://adch.org.uk/, accessed on 16 May 2026) provides minimum welfare and operational standards for shelter/rescue organisations. While these standards highlight and consolidate good overarching principles in shelter animal welfare, their effective translation and embedding into everyday cat care may be difficult to achieve in practice. Additionally, these standards are not legislated, and shelter ADCH membership is voluntary.

Various psychometric methods to assess the behaviour and temperament of cats exist [37], with some specifically developed for, or implemented within, shelter environments [38,39,40,41,42]. However, the applied value of these methods may be limited by various factors, including inadequate demonstration of validity and reliability [43], the presence of anthropocentric and subjective bias [39,44], the ability to be implemented successfully and sustainably by cat care stakeholders [45], in addition to the potential for various contextual factors, including physical health, to influence behavioural presentations [46]. Some assessment protocols developed for cats also include exposing them to stressful and aversive human handling [44,47,48], confinement [41] and novel environments [47], while others demonstrate limited sensitivity and specificity to reliably differentiate between cats in ways that would enable selection of appropriate management pathways for them [49]. Many of the same limitations are relevant to cat welfare and quality of life assessments [50,51,52], making shelter cat management and homing decisions very challenging, especially for cats with complex behavioural and medical needs.

When behaviours such as human-directed fear, aggression and a lack of human sociability are identified in shelter cats, common methods used to try to ‘improve’ a cat’s behaviour and adoptability may inadvertently induce fear, frustration and stress, with questionable long-term benefits. For example, any enforced human proximity and handling that the cat cannot limit their degree of exposure to and/or retreat from represent forms of negative stimulus flooding that remove the cat’s choice, control and autonomy [53,54,55]. The efficacy of these approaches is also questionable, particularly when being attempted with cats already outside, or towards the end of, their more sensitive period for human-socialisation (i.e., 2–7/8 weeks of age [31,56,57]). However, small perceived ‘improvements’ in unsocialised cat’s behaviour during their attempted socialisation may be used as justification for selecting an ultimately inappropriate management pathway for the cat, such as homing them as a ‘pet’ rather than considering alternatives such as TNR/R or euthanasia, leading to poor long-term cat welfare outcomes [31,32,33,34,55].

The nomenclature associated with shelter cat behaviour assessments is also varied and unstandardised. Labels attributed to cats may often rely upon fundamental assumptions about their characteristics and previous lifestyle experiences that cannot be verified or directly measured, but which are used to determine their management pathways and homing outcomes [58,59]. Labels such as ‘stray’ and ‘feral’ as well as ‘socialised’ and ‘sociable’ often mean different things to different cat welfare stakeholders and academics [48,49,56,58,59,60,61,62]. This has the potential to undermine efforts to create consensus around best management practices for cats presenting with various behavioural and demographic characteristics.

Given the substantial short- and long-term welfare implications for cats, it is important that assessments, terminology use, behavioural interventions, management and homing decisions are optimised to support good cat welfare outcomes. However, very little is known about how these processes are currently undertaken by shelters, particularly within the context of the British Isles, including their diversity and consistency of application. The aim of this study was therefore to anonymously survey a range of cat care stakeholders from across the British Isles shelter sector to gain both quantitative and qualitative insight into current approaches to cat assessments, behaviour management, decision-making and homing. This study builds on findings from Part 1 [63], which focused on sector approaches to cat housing and husbandry. Part 1 highlighted positive approaches to meeting cats’ basic needs as well as areas for development, including where cats’ needs could be met more consistently. Collectively, Parts 1 and 2 aim to provide an important baseline of understanding which can be used to holistically identify where good shelter cat care practices are already occurring, and where future interventions and resources could help to better support the sector.

## 2. Materials and Methods

The data collected and analysed within this paper form part of a larger project, and a more detailed version of the study’s methodology is described in Finka et al. [63]. A condensed version containing information relevant to the current study’s aims is provided below.

### 2.1. Ethical Approval, GDPR and Respondent Consent

A favourable ethical opinion was granted from Nottingham Trent University (reference number ARE2223M0404). All respondents were informed about the study characteristics and how their data would be managed before consenting to have their data used for research purposes. Respondents’ personal information (e.g., email address) was anonymised and stored following the current GDPR (Privacy policy|Cats Protection).

### 2.2. Survey Questions

The survey was hosted on a GDPR compliant online platform, and respondents were asked to answer a total of 81 questions, which were grouped into 16 sections. Questions aimed to gather information about relevant cat care practices anticipated to impact shelter cat welfare, based on the current literature, in addition to stakeholder and expert opinion. Questions were informally piloted and refined based on stakeholder feedback (see Part 1 [63] for further detail). Question topics included cat homing assessments, behaviour management and decision-making (covered in the current manuscript) in addition to housing, husbandry, general care and local site operations (addressed in Part 1 [63]). See Supplementary Material for the full list of survey questions; Document S1: Survey Questions. Respondents had the option to skip specific sections and/or select ‘Unsure/don’t know’ responses. Most of the questions were multiple-choice, except for a small number of open-ended and numerical text box answer options. Data from several qualitative questions were also analysed; these covered the specific methods used to determine the suitability of homing cats to different circumstances (i.e., be homed as a pet, with other cats, with dogs) as well as the suitability of prospective adopters to cats.

### 2.3. Survey Eligibility Criteria

To participate in the study, people had to be aged 18 years or over, currently work or volunteer with an animal rehoming/rescue/shelter/sanctuary organisation within the British Isles, and be directly involved in the regular provision of care to cats managed by their organisation. The content of the questions focused predominantly on cat care practices that may vary substantially at local levels (i.e., between cat caregivers, between different sites belonging to the same organisation). Therefore, to maximise overall response rates and support full respondent anonymity, for practical purposes, multiple responses from different individuals within the same shelter organisation were permitted.

### 2.4. Sampling Approach

The sampling period ran from May to October 2023. The survey was launched at the Association of Dogs and Cats Homes (ADCH) 2023 annual conference. Additionally, 189 stakeholders within Cats Protection and 772 separate organisations across the sector were contacted via email or phone and asked to take part in (and disseminate) the survey. To extend its reach, the survey was also shared via Cats Protection’s internal communications channels, via relevant webinars to members of the sector, and in a letter published in the Veterinary Times. Upon survey completion, respondents could opt into a prize draw for the chance to win a £20 voucher for a popular pet accessory store, an online course provided by International Cat Care and an ADCH 2024 conference place.

### 2.5. Data Analysis

Quantitative data were analysed with R Statistical Software 4.5.1 [64] and Microsoft Excel for Microsoft 365 [65], using descriptive summary statistics, including frequencies and percentages for categorical items and mean, median, and range for continuous variables (e.g., years in sector, staff numbers, and shelter capacity). Findings were also interpreted in relation to ADCH minimum standards/recommendations [66] where these were relevant and available and addressed within the discussion. To maintain a focus on broad sectoral trends and avoid non-robust inferential testing, data were primarily reported as a whole, with subgroup descriptives (e.g., volunteer vs. paid hours) provided only for contextual baseline information. Qualitative data (open-ended responses) were analysed using reflexive thematic analysis within a constructionist epistemological framework, with attention to both patterns in the data and their meaningfulness. As part of this process, AMB manually coded the data (without the use of qualitative software) using an inductive open-coding approach. Subsequently, LF worked with AMB to develop overarching topics (i.e., themes) based on patterns identified in the coded data. The themes are reported in the Results section.

To ensure results reflected site-specific operations rather than national or annual totals, extreme outliers representing <2% of responses were excluded: paid staff > 100 (*n* = 6), volunteers > 200 (*n* = 6), and maximum cattery capacity of 1000 cats (*n* = 1). These figures were identified as organisational headcounts or annual throughput inconsistent with the survey’s focal location. Additionally, reported weekly contributions > 90 h (*n* = 6) and individual enclosure measurements ≥ 100 ft (*n* = 2) were removed as probable reporting or unit errors. These exclusions prevented significant skewing of the mean/median, ensuring the data accurately represent typical functional UK cattery environments and individual cat living spaces.

## 3. Results

A brief overview of key respondent characteristics is provided below, with a more detailed summary provided in Finka et al. ([63] in press). In order to provide a holistic overview of approaches to cat assessments and decision-making and reduce the complexity of data reporting as well as to avoid non-robust subgroup comparisons, data were analysed and reported on as a whole rather than as a function of the respondent’s demographics and/or those of their respective shelter organisation. Further information regarding the nature and structure of the sites and organisations respondents were affiliated with can be found in Part 1 [63] and Supplementary Material (S2: Survey Results). A full list of survey questions is included in Supplementary Material (Document S1: Survey Questions).

### 3.1. Respondent Characteristics (n = 393 Total Responses)

A total of 393 unique respondents representing diversity in both site and organisation-level characteristics (see Part 1 [63] and Supplementary Material (S2: Survey Results)) completed the survey, with response rates for individual sections varying between 73 and 100 respondents indicated that they lived with a cat of their own (78.4%, *n* = 308) and that they provided foster care for cats in their private residences while working/volunteering for their current charity/organisation (61.1%, *n* = 240).

Respondents reported working and/or volunteering in the shelter sector for an average of 11.5 years (1–45 years range, median = 8); 56.0% (*n* = 220) reported they were volunteers, 41.2% (*n* = 162) reported that they occupied paid roles, and 2.8% (*n* = 11) mentioned other roles (e.g., shelter owner). Average volunteering hours were 19.1 (1–97 h range, median = 15) per week, and average paid working hours were 35.8 (1–73 h range, median = 35).

In relation to formal qualifications, 34.4% (*n* = 135) of respondents reported having a qualification in animal behaviour, welfare, health, or animal training, and most respondents (89.3%, *n* = 351) reported (relatively recently) undertaking some type of work-based training in support of their current role. Socially interacting with cats was the most commonly reported respondent activity (81.7%, *n* = 321) and assessing the behaviour/welfare of cats (67.7%, *n* = 266) was the least commonly reported.

### 3.2. Nature and Structure of the Organisations (n = 393 Total Responses)

The majority of respondents (68.2%, *n* = 268) reported that their organisation was a member of the Association of Dogs and Cats Homes (ADCH) and that the site where they worked or volunteered was in England (86.0%, *n* = 338) as opposed to other regions of the British Isles.

Most respondents reported that they provided care for cats within a cattery/shelter/rehoming centre (56.5%, *n* = 222) and that this site was part of a wider national organisation that cared for cats across different regions (62.3%, *n* = 245). The most commonly reported cat care activity was the temporary care and housing of cats and their subsequent rehoming (97.5%, *n* = 383). There was an average of 5.6 (0–100 range, median = 2) full-time employees, 3.3 (0–100 range, median = 0) part-time employees and 24.9 (0–200 range, median = 16.5) volunteers per ‘bricks and mortar’ site where the respondent reported providing care for cats.

### 3.3. Cat Behaviour Assessment and Homing

#### 3.3.1. Approaches to the Assessment of a Cat’s Suitability to Be Homed as a Pet/Human Companion (*n* = 332 Total Responses)

The majority of respondents (88.9%, *n* = 295) reported using some formal method to assess a cat’s suitability to be homed as a pet/human companion, while 3.0% (*n* = 10) reported not using any method, and 8.1% (*n* = 27) were unsure or did not know.

Among those who indicated using an assessment method (*n* = 295), the largest percentages reported consistently (i.e., ‘always’) applying these to cats across a range of different cat lifestyles/behaviour categories, where the cat’s suitability to be homed as a pet may be uncertain. However, assessments were less consistently applied to cats deemed to be ‘feral’ compared to other cat lifestyle/behaviour categories; for example, only 57.9% reported ‘always’ assessing feral cats, compared to 85.8% for stray/community cats and 80.5% for previously owned cats with a history of behaviour problems. Additionally, across all categories (except ‘feral’ cats), small percentages of respondents reported infrequently (i.e., ‘never’ or ‘sometimes’) undertaking pet-suitability assessments. See Table 1.

#### 3.3.2. Methods Used to Assess a Cat’s Suitability to Be Homed as a Pet/Human Companion (*n* = 258 Total Responses)

Assessment methods mentioned by respondents included the use of history-based methods, including intake forms (usually completed by the cat’s relinquisher) and observations of the cat’s behaviour and interactions with humans while in the shelter/foster home.

*Intake forms:* Reported examples of information used from the intake forms to aid in the decision-making process included: information about the cat, such as previous history (e.g., if lived with cats or dogs), likes and dislikes (e.g., dislikes children, fears noises), personality (e.g., “nervous cat”), and environmental needs (e.g., outdoor access, a garden).

*Shelter-based observations:* Respondents mentioned that observations were usually performed by carers, fosterers or behaviourists and were used to understand the cat’s level of sociability, particularly how comfortable the cat was around people and if the cat was happy to interact with them. One respondent mentioned that if the cat was considered ‘social,’ it was categorised as a ‘pet’; if the cat was ‘semi-social,’ it was categorised as an ‘outdoor pet’ or ‘barn cat’; and if the cat was ‘not social,’ it was categorised as a ‘feral/TNR’ cat. Some respondents also indicated that veterinary health assessments and on-site observations of the interaction between a cat and their prospective adopter were used to assess the cat’s suitability to be homed as a pet.

#### 3.3.3. Methods Used to Assess a Cat’s Suitability to Be Homed with Other Cats (*n* = 332 Total Responses)

The majority of respondents (82.8%, *n* = 275) reported using an assessment method to determine a cat’s suitability to be homed with other cats, while 7.8% (*n* = 26) indicated that a method was not used and 9.3% (*n* = 31) that they were unsure or did not know.

In the open-ended responses (*n* = 242 total responses), the assessment methods mentioned by respondents were again history-based, including intake forms or conversations about the cat’s previous lifestyle and observations of cat–cat interactions in the shelter or foster home. Reported observation methods ranged from low-intensity, low-intervention to high-intensity ‘test’ exposure scenarios (see further for details).

*Intake forms:* Respondents described that the relinquisher-completed intake form was typically used to determine whether a cat had previously lived with other cats, if it was accustomed to other cats, and the quality of their relationship.

*Shelter-based observations:* Respondents mentioned live observations of cat–cat interactions in both opportunistic and ‘test’ contexts. In opportunistic contexts, the observer (e.g., cat carer, fosterer) described watching the cat’s reaction to the sight, smell, or sound of other cats during ‘chance’ encounters/exposure. In ‘test’ contexts, respondents described creating various situations in which they could assess how the cat would respond when actively exposed to other cats (or cat-related stimuli). Relatively low-intensity/intervention examples mentioned included presenting the focal cat with fabric (e.g., a blanket) containing the scent of the prospective housemate cat (i.e., scent-swapping) and allowing a ‘calm’ cat to walk freely along the corridor while observing how the focal cat reacted inside its pen. More high-intensity/interventional and potentially acute stress-inducing methods included organising a supervised meeting between the focal cat and the prospective housemate cat and putting the focal cat in a large run with other cats.

Some respondents indicated that if the cat was bold/confident or still young (i.e., a kitten), it would usually be deemed suitable to live with other cats. A few respondents also reported that the health of the focal cat would influence the decision-making process, although no further details or context were provided.

#### 3.3.4. Methods Used to Assess a Cat’s Suitability to Be Homed with Dogs (*n* = 332 Total Responses)

The majority of respondents (75.9%, *n* = 252) reported using an assessment method to determine whether a cat could be homed with dogs, while 15.1% (*n* = 50) indicated that no method was used, and 9% (*n* = 30) were unsure or did not know.

As with cat–cat homing assessments, in their open-ended responses (*n* = 224), the assessment methods mentioned by respondents to assess a cat’s suitability to be homed with dogs were history-based, including relinquisher-completed intake forms about the cat’s previous lifestyle and live observations of cat–dog interactions in the shelter/foster home.

*Intake forms:* Respondents described using the intake form to determine whether the cat had previously lived with or been around dogs, whether the relationship was harmonious, and if the cat was generally fearful of dogs.

*Shelter-based observations:* Respondents mentioned live observations of cat–dog interactions in both opportunistic and ‘test’ contexts, with these also varying in intensity and degree of exposure. Opportunistic contexts mentioned included assessing the cat’s reaction to hearing a foster carer’s dog barking. Descriptions of ‘test’ contexts included assessing how the cat responded when presented with dog-related stimuli such as swapping soft furnishings between the cat and a prospective future dog companion. Some respondents mentioned exposing cats to live dogs, including walking a shelter dog past the corridor of the cattery and bringing the prospective companion dog to the shelter to meet the cat.

The age of the cat was again mentioned. Kittens, particularly those less than six months old, were mentioned as being considered suitable for living with dogs. Likewise, respondents indicated that ‘confident’ cats were usually deemed suitable.

#### 3.3.5. Methods Used to Assess a Cat’s Suitability to Certain Domestic Lifestyles (*n* = 332 Total Responses)

When respondents were asked if any method was used to assess the suitability of a cat to certain domestic lifestyles (such as the number of adults and children in a home, ages of children, etc), 85.8% (*n* = 285) indicated that an assessment method was used. Small numbers of respondents reported that no method was used (5.1%, *n* = 17) or that they were unsure if one was used or not (9.0%, *n* = 30).

As with the previous sections, the reported methods within respondents’ open-ended responses (*n* = 241) were history-based, including relinquisher-completed intake forms about the cat’s previous lifestyle, observations of the cat’s behaviour around different people and environments within the shelter, in addition to vet health assessments.

*Intake forms:* Relevant information from the cat’s intake forms mentioned by respondents included the cat’s previous access to the outdoors, experience living with children, pre-existing health conditions and age. Several respondents mentioned that cats who were previously used to having outdoor access were always rehomed to similar environments, except for cats that had certain medical conditions such as FIV, blindness, deafness, etc. In cases where one or more of these conditions were present, respondents mentioned that these cats were typically rehomed as indoor cats. Very elderly cats were also described as usually being rehomed as indoor-only. One respondent mentioned that homing suitability was assessed for all cats except kittens, suggesting that kittens are automatically deemed suitable for a variety of different domestic lifestyles; a sentiment that was also echoed across open-ended responses relating to pet, cat and dog-specific suitability assessments.

*Shelter-based observations:* Respondents reported noting how the cat reacted to different types of social stimuli in the cattery or foster home, including children, adults, and other animals (species unspecified), as well as the cat’s perceived environmental preferences (e.g., preference for indoor pen versus outdoor run). Descriptions of ‘test’ contexts were also mentioned, such as arranging a meeting between the cat and a child from the prospective home prior to adoption or placing the cat in a foster home with children to observe the cat’s response. Respondents also mentioned that the temperament of the cat was considered when determining a cat’s suitability for certain lifestyles. A few respondents mentioned that only ‘confident’ cats were homed with children, and that ‘aggressive’ or ‘nervous’ cats were not. One respondent reported that ‘shy’ cats were rehomed as indoor cats, and another indicated that ‘shy’ cats may be suited to one-person households or those with small numbers of occupants. Finally, one respondent mentioned that pedigree cats were also homed as indoor-only cats.

### 3.4. Behaviour Management of Human-Fearful Cats Within the Shelter

#### 3.4.1. Frequency of Very Fearful Cats Coming into Care (*n* = 333 Total Responses)

When asked how often, over the past 12 months, cats coming into care appeared very fearful of people for more than 48 h post-arrival, the largest percentage of respondents reported that such cats came into care ‘once a month or less’ (36.6%, *n* = 122). This was followed by ‘a few times a month’ (33.3%, *n* = 111), ‘once or twice a week’ (10.5%, *n* = 35), ‘every day or most days’ (3.6%, *n* = 12), and ‘never’ (1.8%, *n* = 6). The remaining respondents (14.2%, *n* = 47) reported they did not know or were unsure.

#### 3.4.2. Approaches to the Behavioural Management of Unfriendly/Fearful/’Aggressive’ (UFA) Cats That Support the Cat’s Autonomy Within the Shelter (*n* = 393 Total Responses)

**Figure 1:**
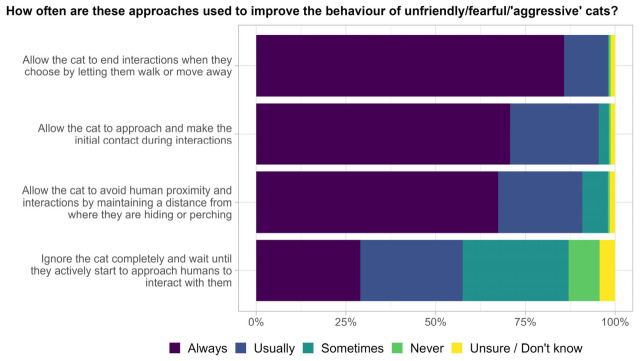
Frequency (and percentage) of approaches applied to ‘improve’ the behaviour of unfriendly, fearful or ‘aggressive’ cats (which support the cat’s autonomy). N = 393 total responses for each item.

Among the approaches used to ‘improve’ the behaviour of UFA cats and/or to encourage them to accept human proximity and/or stroking, the majority of respondents reported they consistently (i.e., ‘always’) used some approaches that supported the cat’s autonomy. For example, 85.8% (*n* = 337) reported ‘always’ allowing the cat to end interactions when they choose, and 70.7% (*n* = 278) reported allowing the cat to approach and make the initial contact. In contrast, ignoring the cat and waiting until it actively approached humans was less consistently applied (29.0%, *n* = 114). Additionally, percentages of respondents (between 0.8% (*n* = 3) and 38.2% (*n* = 150)) reported infrequently (i.e., ‘never’ or ‘sometimes’) applying these autonomy-supporting approaches. See Figure 1.

#### 3.4.3. Approaches to Behavioural Management of Unfriendly/Fearful/’Aggressive’ (UFA) Cats That Do Not Support the Cat’s Autonomy (*n* = 393 Total Responses)

**Figure 2:**
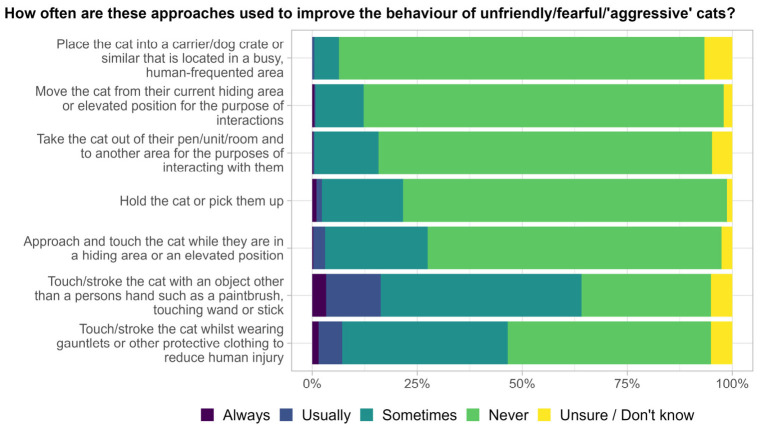
Frequency (and percentage) of approaches applied to ‘improve’ the behaviour of unfriendly, fearful or ‘aggressive’ cats (that do not support the cat’s autonomy). N = 393 total responses for each item.

In terms of behavioural management approaches that would directly compromise the cat’s autonomy, large percentages of respondents reported consistently avoiding (i.e., ‘never’ applying) these methods, for example 87.0% (*n* = 342) reported ‘never’ placing the cat in a carrier/dog crate in a busy area and 85.8% (*n* = 337) ‘never’ moving a cat from their hiding area to facilitate interactions. See Figure 2.

However, some autonomy-compromising methods were less consistently avoided; these included touching/stroking the cat with an object other than a person’s hand, such as a paintbrush, touching wand or stick (30.8%, *n* = 121) and touching/stroking the cat whilst wearing gauntlets (48.3%, *n* = 190). Additionally, across all approaches, small percentages of respondents (0.3%, *n* = 1 to 13%, *n* = 51) reported frequently (i.e., ‘always’ or ‘usually’) applying these methods. See Figure 2.

### 3.5. Cat Homing Outcomes Focusing on Those with Special Requirements

#### 3.5.1. Frequency with Which Cats Deemed Unsocialised and Fearful Are Homed to an Alternative ‘Non-Pet’ Outlet (*n* = 332 Total Responses)

The largest percentages of respondents (from 36.7% *n* *=* 122, to 46.7% *n* *=* 155) reported ‘sometimes’ homing cats deemed unsocialised and fearful of humans to an alternative outlet (e.g., return to the cat’s site of origin, a farm, stables, or a large domestic garden with outdoor food/shelter provided) rather than to a domestic home as a pet (Table 2). This was consistent across different cat age categories (i.e., under 8 weeks, 8 to 16 weeks or over 16 weeks at point of admission). However, younger cats were reportedly less frequently (i.e., ‘always’ or ‘usually’) homed to outlets than older cats.

#### 3.5.2. Outcomes for Cats with Non-Life-Threatening Medical Issues (*n* = 332 Total Responses)

**Figure 3:**
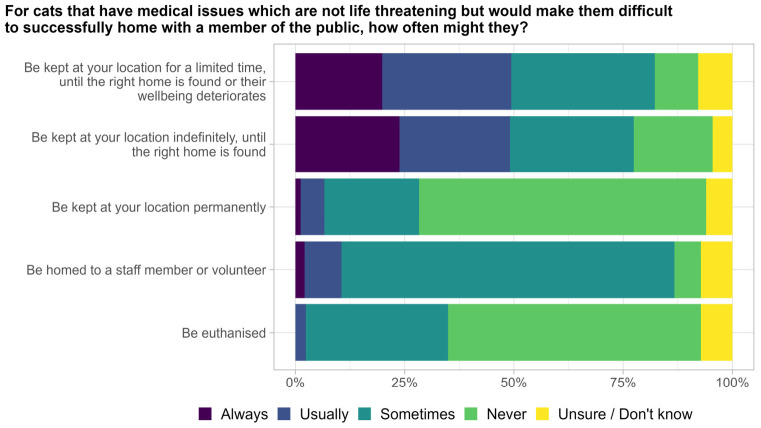
Frequency (and percentage) of different management pathways applied for cats with non-life-threatening medical issues. N = 332 total responses for each item.

Respondents reported on outcomes for cats with medical issues that are not life-threatening but that would make them difficult to successfully home with a member of the public. The majority of respondents reported consistently avoiding (i.e., ‘never’) keeping such cats at the shelter permanently (65.7%, *n* = 218) as well as ‘never’ euthanising them (57.8%, *n* = 192). Most respondents reported ‘sometimes’ homing them to a staff member or volunteer (76.2%, *n* = 253). In relation to housing cats for a limited time, respondents mostly reported either ‘usually’ (29.5%, *n* = 98 ) or ‘sometimes’ (32.8%, *n* = 109) undertaking this, with responses for housing cats indefinitely, relatively balanced across ‘always’ (23.8%, *n* = 79), ‘usually’ (25.3%, *n* = 84) and ‘sometimes’ (28.3%, *n* = 94) response categories. See Figure 3.

#### 3.5.3. Outcomes for Cats Identified as Having Behavioural Issues (*n* = 332 Total Responses)

**Figure 4:**
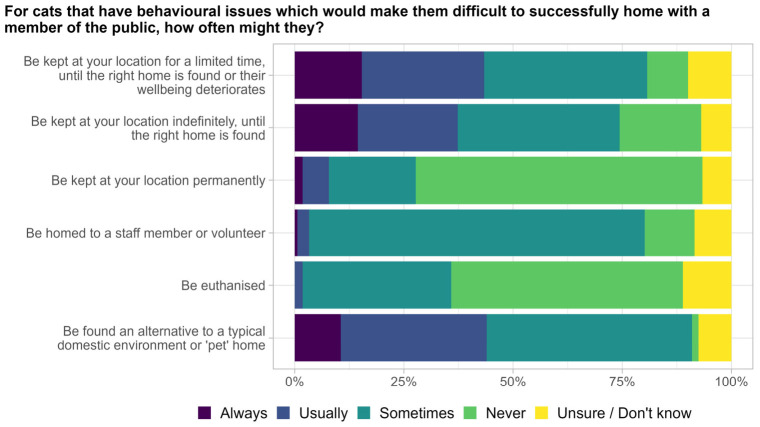
Frequency (and percentage) of management pathways for cats with behavioural issues that make them difficult to home. N = 332 total responses for each item.

Respondents reported on outcomes for cats with perceived behavioural issues that would also make them difficult to successfully home with a member of the public (Figure 4). Similarly to cats with medical issues, the majority of respondents reported consistently avoiding (i.e., ‘never’) keeping such cats at the shelter permanently (65.7%, *n* = 218) as well as ‘never’ euthanising them (53.0%, *n* = 176) and ‘sometimes’ homing them to a staff member or volunteer (76.8%, *n* = 255). In relation to ‘housing cats for a limited time’, respondents mostly reported either ‘always’ (28.0%, *n* = 94) or ‘usually’ (37.3%, *n* = 124) undertaking this, with the highest numbers reporting ‘sometimes’ housing cats indefinitely (37.0%, *n* = 123). Most respondents reported ‘sometimes’ finding an alternative to a typical ‘pet’ home for such cats (47.0%, *n* = 156).

### 3.6. Adopter Suitability Assessments (n = 332 Total Responses)

Nearly all (94.0%, *n* = 312) respondents reported using a formal method to assess the suitability of prospective cat adopters, while 0.9% (*n* = 3) indicated that no method was used and 5.1% (*n* = 17) were unsure or did not know.

In the open-ended responses (*n* = 287), commonly mentioned methods used to gather information about the prospective home of the cat and the adopter/family were application forms, conversations/interviews with the adopter (via phone, internet or in-person) and home visits/checks (virtual or in-person). Other ways of assessing adopter suitability mentioned included street visualisation via online maps, photos of the home, real-life observations of the interaction between prospective adopter and cat in the shelter and vet reference/history (from a previous vet). One respondent also mentioned ‘snooping’ the adopter’s social media accounts.

Types of specific environment based criteria mentioned by respondents included property size and type, if spare rooms were available, if the house was near busy roads, if there was a garden, if the cat would have outdoor access, if a cat flap and outside shelter were available, how busy and noisy the house was, if the adopter rented/owned the house and where the cat’s resources would be placed, etc.

Types of specific adopter/family-related criteria mentioned by respondents included previous experience with cats, what the adopter was looking for in a cat, the age of the household members, if children or other animals lived in the house, if the adopter had a disability, the lifestyle of the adopter/family, etc.

### 3.7. Post-Adoption Follow-Ups (n = 332 Total Responses)

The majority of respondents (63.6%, *n* = 211) reported consistently (i.e., ‘always’) following up with adopters after a cat is homed, while smaller proportions of respondents indicated that follow-ups were ‘usually’ (17.8%, *n* = 59), ‘sometimes’ (13.9%, *n* = 46) or ‘never’ (1.2%, *n* = 4) undertaken, with the remaining indicating they were ‘unsure/did not know’ (3.6% *n* = 12).

## 4. Discussion

### 4.1. General Summary

Building on from Part 1 [63], findings provide important insights into shelter practices across the British Isles regarding current approaches to cat assessments, behaviour management, homing and associated decision-making. Such information provides an important baseline of understanding, from which targeted interventions, resources and support can be developed, and practices refined where needed. This knowledge base and survey framework could also facilitate practical benchmarking and impact assessment via re-sampling, if the shelter sector were to begin to adopt different practices following future regulation. Findings also highlight the need for more detailed sector guidance and support, which could help to ensure that compliance with any future regulations and standards does meaningfully translate into positive welfare experiences for cats. Evidence of good practice, as well as areas where changes may help to enable better cat well-being outcomes, are identified and discussed below. It is advised that the following sections are also considered in the context of the findings and recommendations reported in Part 1 [63], which highlight areas of housing and husbandry practices that could be adapted to better support shelter cats’ holistic well-being and meet their basic needs more consistently.

As is typical with survey designs that rely on self-selection and self-reporting, it is not possible to determine how truly representative or accurate the data and its subsequent inferences are in relation to current sector practices as a whole, particularly considering these at an individual site level. Current findings, however, provide an important starting point from which actionable steps and recommendations to address highlighted issues can be developed and implemented.

### 4.2. Cat Assessments

Survey results indicated that a large proportion of respondents are actively undertaking assessments of admitted cats to determine their suitability to be homed as pets/human companions. This is promising, given the typically diverse spectrum of human sociability within cat shelter populations [47,67] and the negative implications for both cat welfare and the human–animal bond of homing cats to domestic environments when they are not suited to this lifestyle [31]. In relation to the methods described by respondents when undertaking assessments, valuable sources of information gathering, including the use of intake forms and within-shelter behaviour observations, were mentioned. Survey responses would therefore align well with current ADCH requirements [66], which state, ‘*The characteristics of each particular animal shall be assessed in order to identify the most suitable type of home’ and that ‘consideration should be given to the animal’s temperament and behaviour towards people and other animals*’. However, no further details are provided within ADCH guidance in terms of how these assessments should be undertaken regarding cats’ homing suitability and behaviour towards humans or other animals.

Based on the type of information provided by respondents within their open-ended responses, it was unclear the extent to which data-gathering and cat assessment processes were standardised, what they entailed, how the information was processed to inform decision-making, and the relative degree of objectivity/subjectivity involved in each of these steps. There was also a lack of mention of how the well-being of cats was considered during live (potentially acutely stressful) tests and assessments (e.g., those including exposure to other cats and dogs). Further qualitative investigations involving shelter stakeholders would therefore be useful in order to better understand how assessments are undertaken in practice, including their real-time impact on cat well-being.

### 4.3. Terminology Use, Cat-Labelling and Drivers of Decision-Making

In contexts where the cat’s suitability to be homed as a pet may be uncertain, rates at which ‘pet-suitability’ assessments were undertaken on admitted cats varied across different lifestyle/behavioural parameters. Unowned cats from free-roaming environments with either some known or assumed previous interactions with humans (e.g., cats labelled as ‘stray’ or ‘community’) were most consistently assessed. Logically, this makes sense, given individuals from these backgrounds may be particularly varied in terms of their human sociability and often arrive at shelters with either no or very limited behavioural history. Indeed, rather than being clearly differentiable to stakeholders as either ‘pet’ or ‘non-pet’/‘feral’, many cats from ‘stray’ or ‘community’ type backgrounds may occupy relative mid or ‘in-between’ points along a human sociability spectrum [8]. Many of these ‘in-between’ cats may tolerate a degree of social interaction with humans within free-roaming contexts and where humans are a key resource provider, but may not adapt well to human-domestic living under relative confinement. Thus, if cats from ‘stray’ and ‘community’ backgrounds are readily admitted to shelters, careful assessment of each cat is important to ensure that individuals not suited to typical human-domestic living can be identified and alternative ‘non-pet’ homing solutions can be found [8,9].

Interestingly, results suggested that within-shelter behavioural indications of cats’ limited human sociability may be less consistent drivers of ‘pet-suitability’ assessments compared to their pre-existing labels such as ‘stray’ or ‘community cat’. It could be argued that shelter environments have the potential to be very stressful for most cats [24,52,68], and thus a lack of observed human sociability is to be expected. However, when shelter environments apply appropriate stress-management interventions and provide cats with greater autonomy [24,69,70,71,72], human-sociable cats may be more easily differentiated from those that are not. Therefore, if a cat’s needs are sufficiently met within a shelter and they still display a lack of human sociability, this is potentially an indication that they are unsuited to typical human-domestic living, regardless of their previous lifestyle or attributed label.

Additionally, ‘pet-suitability’ assessments were the least consistently applied to cats known (or suspected) by respondents to be ‘feral’. This may appear counterintuitive, given that such cats are least likely to cope well if homed to ‘pet’ environments, and therefore most at risk from unsuitable decision-making and homing outcomes [31]. It is possible that the lower rates of assessment for ‘feral’ cats may reflect the fact that if the cat is presumed to be ‘feral’ prior to, or at the point of, coming into shelter care, a formal pet-suitability assessment may be deemed unnecessary because they are automatically channelled into different management pathways (i.e., TNR/R) compared to those labelled as ‘pet’, ‘stray’ or ‘community’. However, given the lack of standardisation concerning how terms such as ‘feral’, ‘pet’, ‘stray’ and ‘community’ are applied to cats [59], this still raises the question of the types of processes and information sources stakeholders use to determine if a cat is ‘feral’ or not in the first place, and how valid and reliable these methods are.

When describing the assessment of cats based on observations of their behaviour within the shelter or foster home, respondents discussed how this prompted cats to be assigned labels such as ‘shy’, ‘nervous’, ‘confident’, ‘aggressive’ or ‘social’, ‘semi-social’, ‘not social’ or ‘feral’; although details of specific behavioural indicators to support these categorisations were not mentioned. These labels were then linked to further types of categorisation and associated management pathways, including ‘pet’, ‘indoor only’, ‘outdoor pet’, ‘barn cat’, ‘TNR/R’, and ‘not homed to families with children’. While these types of categorisations may be useful in supporting stakeholder decision-making and more individualised approaches to cat homing, the general lack of standardisation regarding terminology and associated assessment processes [12,59] may also limit their benefits in certain situations and to certain cats. For example, cats originating from unowned free-roaming backgrounds, where they have had some degree of human association via supplemental feeding, are commonly admitted to shelters and labelled as ‘strays’. If such cats are not overly sociable towards humans but also do not display human-directed aggression within a shelter environment, they might be labelled as ‘shy’ but ‘social’ by their assessor and homed as a ‘pet’ and potentially ‘indoors-only’. However, if the same cat behaved aggressively, it may be more likely to be labelled as ‘aggressive’ or ‘nervous’ and potentially ‘feral’ or ‘not social’ and subsequently homed to a non-pet outlet (which may actually be the most suitable environment for them).

In addition to the cat being labelled as ‘shy’, the presence of specific medical conditions such as FIV, blindness and the cat being old were also mentioned as grounds for homing the cat to indoor-only domestic environments, irrespective of the cat’s potential lifestyle preferences and previous experiences. These are additional examples of where certain characteristics of cats that are used as the predominant drivers of humans’ decision-making may lead to housing outcomes that may not meet the intrinsic environmental and behavioural needs of the individual cat [73,74].

In general, more careful, consistent and holistic approaches to cat assessment processes, labelling and terminology, and cat-centric decision-making would help to facilitate better cat welfare outcomes at the individual level. The development of nationally agreed, practically operationalised terms to support more standardised approaches to cat terminology use may be useful. However, as discussed, care is advised to ensure that any labels or terms attributed to cats ultimately help rather than hinder their individual management. Alongside the standardisation of terms, it is therefore recommended that guidance and associated training be developed to increase awareness and understanding of cat temperament and lifestyle spectrums [7,8,9]. This should be accompanied by detailed practical support for shelter staff on how to make appropriate admission, management and homing decisions based on relevant cat characteristics, including the cat’s behavioural presentation within the shelter environment.

### 4.4. Methods of Cat and Dog Suitability Assessment

In relation to determining the cat’s suitability to live with other cats, dogs, and within different human-family structures, most respondents reported consistently applying some form of assessment. However, similar to ‘pet’ suitability assessments, based on the nature of information provided by respondents within their open-ended responses, it was unclear how these were undertaken, the way that data were processed to inform decision-making and the relative degree of objectivity/subjectivity involved in each process. There was a lack of mention of how the well-being of cats was considered during assessments, even though some of the methods applied have the potential to cause stress.

Some of the methods of information-sourcing mentioned were retrospective (e.g., history from intake forms) or opportunistic and relatively non-invasive (e.g., observing the cat’s response to hearing dogs barking or when presented with a blanket smelling of an unknown cat). However, respondents also mentioned exposing cats to live ‘test’ scenarios that would be likely to induce acute stress (e.g., putting the cat in a pen with other unknown cats or bringing the prospective adopter’s cat or dog to meet the cat in the shelter).

This is concerning from a cat well-being perspective, particularly in the context of the many other intrinsic risks posed to shelter cats, including disease [20,21,22], general husbandry and handling conditions [23,24,25,26,27]. These forms of high-stress, snapshot sampling methods may also have limitations regarding their sensitivity, specificity and ultimate value as reliable predictors of shelter animals’ behaviour in very different future contexts [75,76].

The development of detailed sector guidance and associated practical training is recommended in order to ensure methods of cat assessments protect cat well-being and equally produce results that enable effective cat homing outcomes and decision-making.

### 4.5. Approaches to Cat Behavioural Management

A proportion of respondents reported regularly admitting cats to their sites that appeared persistently human-fearful. In relation to the behavioural management/modification of cats considered fearful and/or behaving aggressively, most respondents reported consistently applying some approaches that would support the cat’s autonomy (e.g., allowing the cat to approach and make the initial contact during interactions). However, the application of methods in direct contrast to this was also frequently reported by some respondents (e.g., holding the cat or picking them up, approaching and touching the cat while they are hiding, touching them with a paintbrush, or whilst wearing gauntlets). These latter types of human–cat interactions can often be included under the umbrella term of ‘behavioural modification’ and assumed to support a cat’s ‘desensitisation’ and ‘counter-conditioning’ to humans. However, the reality is that such processes are likely to induce acute negative responses in cats that already struggle with human contact, thus providing limited (or no) benefits to their well-being [54] and future ability to cohabit with humans [31].

ADCH minimum standards stipulate “All training and behaviour modification shall be done through positive reinforcement methods without the use of coercive or punitive techniques and/or equipment. This precludes the use of intimidation, physical punishment and fear as well as the use of any equipment that may cause pain and fear which will compromise the welfare of the animal.” However, examples of methods that may cause pain and fear, or guidance on how to determine when an animal’s welfare is compromised, are not provided. Additionally, no specific guidance is provided in relation to undertaking behavioural modification or human–cat interactions in general, and how to avoid compromising the cat’s well-being in the process. Thus, while the above methods of cat behavioural management have the potential to induce fear, it is likely difficult to establish in practice whether these processes would be in contradiction to ADCH regulations. The development of detailed sector guidance and associated practical training would be helpful to ensure more consistent cat-autonomy-focused approaches to human–cat interactions. It is also recommended that this be accompanied by an appropriate decision-making process to help determine whether ‘behavioural modification’ approaches are suitable to attempt with persistently fearful cats, or if homing to alternative ‘non-pet’ outlets could be a more appropriate outcome.

### 4.6. Cat Homing Outcomes

In relation to reported homing outcomes for cats deemed to be unsociable and persistently human-fearful (key traits commonly ascribed to cats deemed ‘feral’ [58,59]), individuals were not consistently homed to ‘alternative’ or ‘non-pet’ outlets (e.g., free-roaming locations such as farms, barns, rural areas). While older cats (particularly those aged over 4 months) were more consistently homed to ‘alternative’ outlets compared to those under 4 months, this still leaves large proportions of unsocialised cats already towards the end of, or well outside of, their optimal human–socialisation window (i.e., from 2 to 7/8 weeks of age [56]), potentially being homed as ‘pets’. These findings are concerning, given the limited plasticity for positive human–social learning in fearful, unsociable cats [31,56,77,78,79] and the short and long-term welfare consequences of their enforced close cohabitation with humans [10,31]. Indeed, these findings would appear to be in contradiction to current ADCH guidelines, which state, “*Adult feral cats are not suitable to be kept as companion animals. In some cases, kittens born from feral adults may be suitable to be kept as companion animals if they are socialised towards humans for the entirety of their socialisation period (i.e., 2–7/8 weeks of age). It may be possible to begin socialising feral kittens older than 2 weeks of age if they do not appear fearful or distressed in the presence of humans and they are still under the age of 8 weeks*”. Reasons for the inconsistent homing of persistently human-fearful cats to ‘alternative’ outlets were not explored within the survey. However, greater shelter resource investments in schemes to proactively secure various types of ‘non-pet’ homing options in advance of when they are needed may help to encourage greater access to, and use of, these options where appropriate.

For cats deemed to have any type of behavioural or non-life-threatening medical issue that would make them difficult to successfully home with the public, respondents’ answers suggested that many of these cats could face a prolonged, indefinite or even permanent stay within a shelter. This is also concerning, given that shelter environments pose serious welfare risks to cats and that longer shelter stays are associated with increased risks [20,21,22,24,26,27]. Depending on the specific behavioural and medical issues in question, if these are sufficient to limit a cat’s ease of homing to the public, this may also mean that the cat has a poor prognosis for long-term quality of life in general, or at least not without intensive human management. Homing of such cats to staff and volunteers was frequently reported, and while this may enable certain cats to receive more specialised care to support their well-being, this may not always be appropriate or sufficient to guarantee a good quality of life [80]. Reported frequencies with which such cats would be euthanised rather than kept on site or homed were very low. However, in certain cases, euthanasia might be the best welfare outcome to prevent future suffering and poor quality of life [81,82], as well as to support effective use of shelter resources. In combination with greater efforts to provide cats with access to homing options and outcomes that better meet their needs (i.e., alternatives to ‘pet’ homes), more pragmatic discussions within the shelter sector about the role of euthanasia as an important welfare intervention to prevent poor quality of life may be useful.

### 4.7. Prospective Adopter Assessments, Criteria and Follow-Ups

Almost all respondents consistently reported undertaking suitability assessments of prospective adopters. A range of relevant factors covering the adopters’ previous experience of cat ownership, in addition to their current physical and social home environment, were mentioned as important criteria for decision-making. However, as with cat assessments, it was unclear how standardised these data-gathering processes were, how information was processed to inform decision-making, and the degree of objectivity/subjectivity involved in each of these steps. Despite the lack of specific details provided, the processes mentioned did appear to align with relevant ADCH guidance which includes “*Information about the prospective new owner/keeper and their family shall be obtained to ensure their circumstances and facilities fit the requirements of the particular animal they wish to rehome*” and “*It is good practice to interview the prospective new keeper and complete a re-homing application form which should request information about previous experience of animal ownership, the lifestyle of the family and the facilities which would be on offer at the new home*”.

Interestingly, traditional approaches to shelter animal adoption have been criticised for their potentially restrictive, overly subjective or non-evidence-based approaches [83,84]. The potential implications of this are animals’ limited accessibility to interested adopters, leading to longer lengths of stay and overall greater drain on shelter resources. However, a robust scientific evidence base of the types of human and environmental characteristics that are most/least important to successful animal adoption is lacking [84]. Therefore, pragmatic approaches to adopter assessments that also prioritise the individual needs of specific cats are likely to lead to the best balance between finding the perfect homing option and the time cats must spend in a shelter. In support of this, systematic post-adoption follow-ups may be particularly helpful in filling current data gaps; identifying which characteristics of cats, humans and data-gathering methods are most helpful to facilitate positive long-term homing outcomes for cats, as well as highlighting where assessments and matching processes could be refined. Follow-ups can also help to ensure that new adopters are sufficiently supported to help their adopted cat thrive in its new environment.

However, only slightly more than half of respondents reported consistently undertaking post-adoption follow-ups for cats. Although the survey did not provide an option for respondents to explain why, this could be linked to the limited resources and current financial strains within the sector [85,86,87,88]. As highlighted by findings reported in Finka et al. 2026 [63], greater emphasis on quality of care provision rather than capacity [17,89] may be helpful to ensure shelters are only admitting as many cats as they are able to provide good standards of care (and aftercare) to, based on the current resources available. Further guidance and support for the sector in terms of how to undertake effective post-adoption follow-ups may also be beneficial.

### 4.8. Study Limitations

Limitations associated with the current study design, including true sector representativeness, accuracy of data obtained via self-selection and self-reporting methods and direct links between study findings and cat welfare are discussed in detail in Part 1 [63]. To address these limitations, future studies could request that survey respondents forgo their anonymity so that individual sites/organisations could be identified and accounted for in data summary calculations and for data collection to incorporate in-person data sampling of individual sites and shelter cat populations. However, such approaches would require much greater resources to facilitate and/or could potentially present conflicts of interest, given that shelter organisations and their stakeholders may feel reluctant to participate if there is the potential for them to feel personally targeted.

## 5. Conclusions and Recommendations

Survey responses indicate that overall, shelter cat caregivers are consistently undertaking cat and prospective adopter assessments, and that the information gathered during these processes is being used to support decision-making and cat homing outcomes. However, approaches appeared varied and unstandardised, and in the absence of detailed, evidence-informed guidance, it is possible that these processes do not always translate to good cat well-being outcomes at the individual level. This research is timely given the substantial interest in future sector regulation, including the UK government position regarding consideration of the need for additional guidance and regulatory actions, and the associated parliamentary bill currently under debate [90,91].

To ensure cats are behaviourally managed, assessed and homed appropriately, it is recommended that greater consideration is placed on cats’ within-shelter behavioural presentations as grounds for pragmatic pet-suitability assessments. Such assessments will be more effective if used in combination with enhanced efforts to appropriately meet the cat’s needs and manage cat stress levels within a shelter environment [24,69,70,71,72]. Careful attention should also be taken to ensure that methods of behavioural assessment and management applied within the shelter environment are appropriate to their intended outcomes, and do not negatively impact on cat well-being during their implementation. More careful and consistent application of assessment processes, cat-labelling and terminology use are recommended to ensure cat well-being friendly decision-making and outcomes. Prioritising the sourcing of alternative homing options for cats not suited to human-domestic living, in addition to pragmatic decision-making, including euthanasia to prevent long stays, poor quality of life outcomes and shelter resource burden are also recommended.

Given the predominant absence of any legal regulation or licensing of the British Isles shelter sector, or sufficiently detailed guidance, training and accessible support for shelter workers and volunteers, a clear pathway for these improvements to be actioned at scale is currently lacking. To facilitate positive change, beneficial steps could therefore include a move towards greater sector regulation and guidance. It is recommended that this occurs in combination with the development of appropriate shelter training, resources and support, and associated evaluation and impact assessments in order to ensure regulations and guidance translate into practical, evidence-based benefits to shelter stakeholders and cat welfare. To this effect, large-scale repeated sector surveying to compare pre and post-licencing approaches to cat assessments and homing would also be useful.

### Suggested Resources to Support Approaches to Cat Assessments, Behavioural Management and Homing Decisions

The development of guidance and associated resources in the following areas is recommended to ensure consistent positive organisation and site-level approaches:

• The creation of nationally agreed, operationalised terms to support standardised approaches to cat terminology, with associated training and resources to support their practical and appropriate application.
• Detailed sector guidance, training and practical support for shelter staff on appropriate, cat-friendly methods to assess cat suitability to be ‘pets’, live with other cats and dogs, etc.
• Detailed sector guidance, training and practical support for shelter staff on how to make appropriate admission and homing decisions based on relevant cat characteristics, including the cat’s behavioural presentation within the shelter environment.
• Detailed sector guidance and practical training to support more consistent cat-autonomy-focused approaches to human–cat interactions, including decision-making around the appropriateness of and approaches to ‘behavioural modification.’
• Sector guidance and support around pragmatic decision-making in relation to cat homing and outcomes, including the important role of ‘alternative’ homing options and euthanasia to prevent long-term poor quality of life and limit shelter resource burden.
• Schemes to support local shelter access to various homing options for cats with specialised needs, e.g., ‘alternative’ or ‘non-pet’ outlets for fearful, unsociable cats.
• Sector guidance and support in undertaking effective cat-adopter assessments and post-adoption follow-ups, and how to use this information to refine future processes.

## Figures and Tables

**Table 1:** Frequency (and percentage) with which methods were applied to different cat populations to assess their suitability to be homed as a pet/human companion. DK = Do not know.

Category	Never	Sometimes	Usually	Always	Unsure/DK/NA	Total
Stray or community cats	*n* = 1 0.3%	*n* = 10 3.4%	*n* = 28 9.5%	*n* = 248 84.1%	*n* = 8 2.7%	*n* = 295
Previously owned cats where the person relinquishing the cat provides information suggesting the cat exhibited behaviour problems in the previous home	*n* = 2 0.7%	*n* = 16 5.4%	*n* = 35 11.9%	*n* = 235 79.7%	*n* = 7 2.4%	*n* = 295
Cats that appear to be experiencing high stress levels and generally failing to cope whilst housed at your location	*n* = 18 6.1%	*n* = 25 8.5%	*n* = 21 7.1%	*n* = 222 75.3%	*n* = 9 3.1%	*n* = 295
Cats that exhibit aggressive behaviour towards staff or volunteers whilst housed at your location	*n* = 13 4.4%	*n* = 31 10.5%	*n* = 21 7.1%	*n* = 221 74.9%	*n* = 9 3.1%	*n* = 295
Cats that exhibit fearful behaviour towards staff or volunteers whilst housed at your location	*n* = 9 3.1%	*n* = 34 11.5%	*n* = 26 8.8%	*n* = 220 74.6%	*n* = 6 2.0%	*n* = 295
Cats that do not seem to actively enjoy interactions with staff or volunteers (i.e., they may tolerate interactions but tend not to solicit them)	*n* = 17 5.8%	*n* = 29 9.8%	*n* = 31 10.5%	*n* = 211 71.5%	*n* = 7 2.4%	*n* = 295
Feral cats or those suspected to be feral	*n* = 57 19.3%	*n* = 38 12.9%	*n* = 12 4.1%	*n* = 151 51.2%	*n* = 37 12.5%	*n* = 295

**Table 2:** Frequency (and percentage) with which unsociable and fearful cats are homed to an ‘alternative outlet’ rather than to a domestic home as a pet, according to the age of the cat on arrival at the shelter. N = 332 total responses for each item. DK = Do not know.

Age of the Cat on Arrival to Shelter	Never	Sometimes	Usually	Always	Unsure/DK	Total
Under 8 weeks old	*n* = 113 34.0%	*n* = 122 36.7%	*n* = 41 12.3%	*n* = 24 7.2%	*n* = 32 9.6%	*n* = 332
8 to 16 weeks old	*n* = 54 16.3%	*n* = 155 46.7%	*n* = 57 17.2%	*n* = 28 8.4%	*n* = 38 11.4%	*n* = 332
Over 16 weeks old	*n* = 20 6.0%	*n* = 130 39.2%	*n* = 84 25.3%	*n* = 74 22.3%	*n* = 24 7.2%	*n* = 332

## Data Availability

The data presented in this study are openly available in the Open Science Framework at https://osf.io/x4gmf/?view_only=92148f18672f49c29281bd5fc786322d (accessed on 16 May 2026).

## Supplementary Materials

The following supporting information can be downloaded at: https://www.mdpi.com/article/10.3390/vetsci13060590/s1, Document S1: Survey Questions; Document S2: Survey Results.

## Abbreviations

The following abbreviations are used in this manuscript:

UK	United Kingdom
ADCH	Association of Dogs and Cats Home
TNR/R	Trap-Neuter-Return/Relocate
GDPR	General Data Protection Regulation
FIV	Feline Immunodeficiency Virus
UFA	Unfriendly/fearful/aggressive
