# Supplementary material for: Part 2: A Sector-Wide Survey of UK/British Isles Shelter Organisations Caring for Cats: Caregiver-Reported Approaches to Assessments, Behaviour Management and Homing Decisions
Source: Vet Sci. 2026 Jun 18;13(6):590. doi: 10.3390/vetsci13060590 (PMC13307826; doi:10.3390/vetsci13060590)
Supplement: Supplementary file 1 [file vetsci-13-00590-s001.zip › vetsci-4038911_Document S2 - Survey Results.pdf]

Document S2: Survey Results

Survey responses by section

Section	n	Percent
Section 1: Details of the charities/organisations and participants roles	393	100%
Section 2: Numbers of staff and volunteers at your location	345	87.8%
Section 3: Numbers of cats, pens and cats per staff at the site	347	88.3%
Section 4: Cat intake/admissions and waiting lists	337	85.8%
Section 5: Environmental provisions within cat pens/units	393	100%
Section 6: Moving of cats between different pens/units	344	87.5%
Section 7: Details about single and multi-cat housings	364	92.6%
Section 8: Average sizes of single and multi-cat pens/units	290	73.1%
Section 9: Preventive health care and vet checks	368	93.6%
Section 10: Medicating cats and handling for health checks	346	88.3%
Section 11: Cleaning cat pens/units	361	91.9%
Section 12: Cat socialisation, interactions and behaviour management	393	100%
Section 13: Cat rehoming assessments and processes	332	84.5%
Section 14: Assessing and managing cat wellbeing	333	84.7%
Section 15: Participants' perspective on cat wellbeing tools and necessary support	393	100%
Section 16: Participant demographics	393	100%

Q80. How many of your *own cats* currently live with you?

Response	Number of kittens/cats	n	Percent
Kittens ages aged 16 weeks or under	0	302	76.8%

	1	5	1.3%
	2	1	0.3%
Cats over 16 weeks of age	0	2	0.5%
	1	116	29.5%
	2	77	19.6%
	3	38	9.7%
	4	19	4.8%
	5	15	3.8%
	6	12	3.1%
	7	10	2.5%
	8	6	1.5%
	9	6	1.5%
	10	2	0.5%
	12	2	0.5%
	14	2	0.5%
	21	1	0.3%
I don’t currently live with any cats or kittens		85	21.6%

Note: Percentages are based on 393 non-missing responses.

Q4. Do you currently provide or have you previously provided foster care for cats within your private residence whilst working or volunteering for your current charity/organisation?

Response	n	Percent
Yes	240	61.1%
No	153	38.9%

Note: Percentages are based on 393 non-missing responses.

Q 79. Please indicate the number of active years in total you have either worked and/or volunteered within the rehoming/shelter/rescue sector

Response	n	Percent
1	41	10.57%
2	27	6.96%
3	19	4.90%
4	23	5.93%
5	25	6.44%
6	19	4.90%
7	20	5.15%
8	24	6.19%
9	10	2.58%
10	20	5.15%
11	6	1.55%
12	17	4.38%
13	8	2.06%
14	12	3.09%
15	17	4.38%
16	6	1.55%
17	2	0.52%
18	8	2.06%
19	1	0.26%
20	13	3.35%
21	3	0.77%
22	6	1.55%

Response	n	Percent
23	7	1.80%
24	4	1.03%
25	14	3.61%
26	2	0.52%
28	3	0.77%
29	1	0.26%
30	11	2.84%
32	1	0.26%
34	1	0.26%
35	6	1.55%
36	2	0.52%
37	1	0.26%
40	4	1.03%
42	1	0.26%
43	1	0.26%
44	1	0.26%
45	1	0.26%

Note: Percentages are based on 388 non-missing responses.

Q1. Is your primary role within your charity/organisation?

Response	n	Percent
A paid position	162	41.22%
A voluntary position	220	55.98%
Other (founder/run the sanctuary)	8	2.0%
Other (answer unclear)	2	0.5%

Response	n	Percent
Other (missing response)	1	0.25%

Note: Percentages are based on 393 non-missing responses.

Q2. How many hours a week on average do you work or volunteer for your charity/organisation?

Role	Weekly Hours	n	Percent
Paid	1 - 9	1	0.6%
	9 - 17	3	1.9%
	17 - 25	14	8.7%
	25 - 33	18	11.2%
	33 - 41	104	64.6%
	41 - 49	13	8.1%
	49 - 57	5	3.1%
	57 - 65	1	0.6%
	65 - 73	2	1.2%
Voluntary	1 - 9	68	34.5%
	9 - 17	46	23.4%
	17 - 25	19	9.6%
	25 - 33	32	16.2%
	33 - 41	15	7.6%
	41 - 49	4	2.0%
	49 - 57	6	3.0%
	57 - 65	3	1.5%
	65 - 73	1	0.5%
	81 – 89	1	0.5%

Role	Weekly Hours	n	Percent
	89 - 97	2	1.0%
Other	1 - 9	1	11.1%
	9 - 17	0	0.0%
	17 - 25	1	11.1%
	25 - 33	0	0.0%
	33 - 41	3	33.3%
	41 - 49	0	0.0%
	49 - 57	0	0.0%
	57 - 65	0	0.0%
	65 - 73	2	22.2%
	81 – 89	1	11.1%
	89 - 97	1	11.1%

Note: Percentages are based on 367 non-missing responses. Reported weekly hours > 90 were removed (n = 6).

Q81. Do you hold any formal qualifications in animal behaviour, welfare, health or animal training?

Response	n	Percent
Yes	135	34.4%
No	235	59.8%
Prefer not to say	23	5.9%

Note: Percentages are based on 393 non-missing responses.

Q81. Please specify your formal qualifications in animal behaviour, welfare, health or animal training

Response	n	Percent
Masters/level 7 qualification	11	10.4%

BSc/level 6 qualification	32	30.2%
Level 5 qualification	3	2.8%
Level 4 qualification	4	3.8%
Level 3 qualification	18	17.0%
Level 2 qualification	14	13.2%
Diploma at unknown level	15	14.2%
RVN (qualification unknown)	9	8.5%

Note: Percentages are based on 106 non-missing affirmative responses to the question: "Do you hold any formal qualifications in animal behaviour, welfare, health or animal training?"

Q6. Have you undertaken any of the following types of training in support of your current role?

Training	Response	n	Percent
Understanding and meeting cat's basic needs within the shelter environment	Yes	351	89.31%
	No	42	10.69%
Recognising good and also poor welfare in cats in the shelter environment	Yes	318	80.92%
	No	75	19.08%
Understanding and interpreting cat's behaviour and body language	Yes	344	87.53%
	No	49	12.47%
Interacting with and handling cats appropriately	Yes	337	85.75%
	No	56	14.25%
Disease prevention, recognition and outbreak control	Yes	325	82.70%
	No	68	17.30%
Pen and associated equipment cleaning	Yes	312	79.39%
	No	81	20.61%
Unsure/can't remember		2	0.51%

Training	Response	n	Percent
Other		55	29.73%
No training undertaken		21	5.34%

Q3. What cat care activities do you regularly undertake as part of your role?

Activity	n	Percent
Feeding cats	278	70.74%
Cleaning cat pens and associated equipment/areas	282	71.76%
Socially interacting with cats	321	81.68%
Undertaking behaviour and/or welfare assessments of cats	266	67.68%
Implementing cat training or other behavioural modification	181	46.06%
Providing support to other care staff/volunteers in the form of cat behaviour and/or welfare advice	259	65.90%
Managing or supervising individuals that provide regular care for cats	212	53.94%
Training individuals that provide regular care for cats	181	46.06%
Involved in cat intake/admission	261	66.41%
Involved in cat adoption/rehoming	290	73.79%
Kitten hand rearing	140	35.62%
Other (health)	26	6.6%
Other (fostering)	9	2.3%

Activity	n	Percent
Other (TNR/trapping)	13	3.3%
Other (transporting cats)	6	1.5%
Other (social media)	4	1.0%
Other (help line/advice)	7	1.8%
Other (administration)	11	2.8%
Other (fundraising/finances)	10	2.5%

Note: Percentages are based on 393 respondents. Multiple selections allowed.

Q12. Is your charity/organisation a member of the Association of Dogs and Cats Homes (ADCH)?

Response	n	Percent
Yes	268	68.2%
No	71	18.1%
Unsure/don't know	54	13.7%

Note: Percentages are based on 393 non-missing responses.

Q10. Where is the place that you care for cats located?

		**Reported location of the respondent's site**		**Location of all identified/contacted cat shelter organisations/ individual branches and centres across the British Isles**	
Response		n	Percent	n	Percent
England	North East	15	3.8%	39	3.6%
	North West	33	8.4%	118	11.0%
	Yorkshire and the Humber	26	6.6%	82	7.6%

	West Midlands	20	5.1%	93	8.7%
	East Midlands	29	7.4%	81	7.6%
	East England	40	10.2%	120	11.2%
	London	47	12.0%	52	4.9%
	South East	69	17.6%	165	15.4%
	South West	59	15.0%	103	9.6%
Wales	North	9	2.3%	15	1.4%
	Mid/Central	2	0.5%	10	0.9%
	South	8	2.0%	34	3.2%
Scotland	Highlands and Islands	3	0.8%	15	1.4%
	Grampian	3	0.8%	8	0.7%
	Lothian and Borders	2	0.5%	8	0.7%
	Tayside	3	0.8%	5	0.5%
	Fife	0	0.0%	7	0.7%
	Strathclyde	3	0.8%	15	1.4%
	Central Scotland	3	0.8%	7	0.7%
	Dumfries and Galloway	0	0.0%	3	0.3%

Republic of Ireland	Connacht/ Connaught	2	0.5%	11	1.0%
	Leinster	2	0.5%	34	3.2%
	Munster	0	0.0%	14	1.3%
	Ulster	0	0.0%	5	0.5%
Northern Ireland		12	3.1%	21	2.0%
Isle of Man or UK Channel Islands (E.g. Guernsey, Jersey, Alderney, Herm and Sark)		3	0.8%	7	0.7%
Unsure/don't know		0	0.0%	0	0.0%
Prefer not to say		0	0.0%	0	0.0%

Note: Percentages are based on 393 non-missing responses.

Q9. Is this main place where you care for cats:

Response	n	Percent
Part of a wider **national** organisation including multiple sites/catteries/branches/centres that care for cats across different regions	245	62.3%
Part of a wider **local** organisation including multiple sites/catteries/centres that care for cats within a specific region	82	20.9%
Independent (i.e. a single site) and **not part of a wider national or local organisation**	48	12.2%
Other (Please provide some brief details)	16	4.1%
Unsure/don't know	2	0.5%

Note: Percentages are based on 393 non-missing responses.

Q8. Which of the following best describes the main environment where you provide care for cats within your organisation?

Response	n	Percent
A cattery/shelter/rehoming centre (separate from a private domestic residence)	222	56.5%
A **collection of pens** near to or adjoining a private residence (i.e. in a private garden or built as an extension to a private home)	49	12.5%
A **single pen** near to or adjoining a private residence (i.e. in a private garden or built as an extension to a private home)	27	6.9%
A room or rooms within a private domestic residence	90	22.9%
Other<br>(Please provide some brief details)	5	1.3%

Note: Percentages are based on 393 non-missing responses.

Q11. What kinds of services are provided at this location?

Response	n	Percent
Temporary care and housing of shelter/rescue cats and their subsequent rehoming to members of the public	383	97.5%
Permanent care and housing of cats	51	13.0%
Temporary care and housing of cats (for commercial purposes such as paid boarding for privately-owned cats)	16	4.1%

Trap neuter return of unowned (i.e. stray/feral/street/community) cats	167	42.5%
Trap neuter relocate of unowned cats	138	35.1%
Neutering of owned cats within the community	125	31.8%
Temporary care and housing of other species	104	26.5%
Permanent care and housing of other species	23	5.9%

Note: Percentages are based on 393 respondents. Multiple selections allowed.

Q13. How many people are:

Paid Staff Members	*n*	*Percent*	*Part-Time Staff*	*n*	*Percent*	*Volunteers*	*n*	Percent
0	129	32.8%	0	138	35.1%	0	5	1.3%
1-10	108	27.5%	1-10	122	31.0%	1-20	163	41.5%
11-20	29	7.4%	11-20	12	3.1%	21-40	63	16.0%
21-30	10	2.5%	21-30	2	0.5%	41-60	30	7.6%
31-40	1	0.3%	31-40	0	0.0%	61-80	10	2.5%
41-50	2	0.5%	41-50	0	0.0%	81-100	5	1.3%
51-60	0	0.0%	51-60	0	0.0%	101-120	2	0.5%
61-70	1	0.3%	61-70	0	0.0%	121-140	1	0.3%
71-80	1	0.3%	71-80	0	0.0%	141-160	0	0%

81-90	0	0.0%	81-90	0	0.0%	161-180	0	0%
91-100	1	0.3%	91-100	1	0.3%	181-200	3	0.8%
Don’t know	107	27.2%	Don’t know	116	29.5%	Don’t know	105	26.7%

Note: Percentages are based on 393 respondents

Q21. Are any assessment methods or criteria used to determine whether a cat is suitable to admit/bring into care or not?

Response	n	Percent
Yes	235	69.73%
No	44	13.06%
Unsure/don’t know	58	17.21%

Note: Percentages are based on 337 non-missing responses.

Q51. Are any assessment methods or criteria used to determine whether a cat is suitable to be homed as a pet/human companion?

Response	n	Percent
Yes	295	88.9%
No	10	3.0%
Unsure/don’t know	27	8.1%

Note: Percentages are based on 332 non-missing responses.

Q52. Are any assessment methods or criteria used to determine whether a cat is suitable to be homed with other cats?

Response	n	Percent
Yes	275	82.8%
No	26	7.8%
Unsure/don’t know	31	9.3%

Note: Percentages are based on 332 non-missing responses.

Q52. Are any assessment methods or criteria used to determine whether a cat is suitable to be homed with dogs?

Response	n	Percent
Yes	252	75.9%
No	50	15.1%
Unsure/don’t know	30	9.0%

Note: Percentages are based on 332 non-missing responses.

Q52. Are any assessment methods or criteria used to assess a cat’s suitability to certain domestic lifestyles (i.e. indoor versus outdoor, number of adults/children in a home, ages of children etc)?

Response	n	Percent
Yes	285	85.8%
No	17	5.1%
Unsure/don’t know	30	9.0%

Note: Percentages are based on 332 non-missing responses.

Q58. Over the past 12 months or so, on average, how often would you say cats (including kittens) that come into care appear consistently very fearful* of people? (i.e. fearful for more than 48 hours post arrival)?

Response	n	Percent
Never	6	1.8%
Once a month or less	122	36.6%
A few times a month	111	33.3%
Once or twice a week	35	10.5%
Every day or most days	12	3.6%

Response	n	Percent
Unsure/don't know	47	14.1%

*Note: Percentages are based on 333 non-missing responses. *A cat that is fearful in people's presence might be constantly or frequently hiding or attempting to hide, appear frozen, try to flee or escape and/or hiss, growl, bite or swipe when approached or touched)*

Q47. In general, for physically healthy cats that appear unfriendly/fearful/behave aggressively, what approaches might be used to try to improve their behaviour and/or encourage them to accept human proximity/being stroked?

Approach	Response	n	Percent
Approach and touch the cat while they are in a hiding area or an elevated position	Never	275	70.0%
	Sometimes or occasionally	96	24.4%
	Usually	11	2.8%
	Always	1	0.3%
	Unsure/don't know	10	2.5%
Move the cat from their current hiding area or elevated position for the purposes of interactions	Never	337	85.8%
	Sometimes or occasionally	45	11.5%
	Usually	1	0.3%
	Always	2	0.5%
	Unsure/don't know	8	2.0%
Hold the cat or pick them up	Never	303	77.1%
	Sometimes or occasionally	76	19.3%
	Usually	5	1.3%
	Always	4	1.0%
	Unsure/don't know	5	1.3%

Touch/stroke the cat with an object other than a person’s hand such as a paintbrush, touching wand or stick	Never	121	30.8%
	Sometimes or occasionally	188	47.8%
	Usually	51	13.0%
	Always	13	3.3%
	Unsure/don't know	20	5.1%
Touch/stroke the cat whilst wearing gauntlets or other protective clothing to reduce human injury	Never	190	48.3%
	Sometimes or occasionally	155	39.4%
	Usually	22	5.6%
	Always	6	1.5%
	Unsure/don't know	20	5.1%
Place the cat into a carrier/dog crate or similar that is located in a busy, human-frequented area	Never	342	87.0%
	Sometimes or occasionally	23	5.9%
	Usually	2	0.5%
	Always	0	0.0%
	Unsure/don't know	26	6.6%
Allow the cat to approach and make the initial contact during interactions	Never	2	0.5%
	Sometimes or occasionally	11	2.8%
	Usually	97	24.7%
	Always	278	70.7%

	Unsure/don't know	5	1.3%
Allow the cat to end interactions when they choose by letting them walk or move away	Never	2	0.5%
	Sometimes or occasionally	1	0.3%
	Usually	48	12.2%
	Always	337	85.8%
	Unsure/don't know	5	1.3%
Allow the cat to avoid human proximity and interactions by maintaining a distance from where they are hiding or perching (if in an elevated position)	Never	2	0.5%
	Sometimes or occasionally	28	7.1%
	Usually	92	23.4%
	Always	265	67.4%
	Unsure/don't know	6	1.5%
Ignore the cat completely and wait until they actively start to approach humans to interact with them	Never	34	8.7%
	Sometimes or occasionally	116	29.5%
	Usually	112	28.5%
	Always	114	29.0%
	Unsure/don't know	17	4.3%
Take the cat out of their pen/unit/room and to another area for the purposes of interacting with them	Never	312	79.4%
	Sometimes or occasionally	60	15.3%

	Usually	1	0.3%
	Always	1	0.3%
	Unsure/don't know	19	4.8%
Move the cat to a more suitable pen/unit/room in order to provide them with living quarters that are larger or in a quieter area	Never	51	13.0%
	Sometimes or occasionally	184	46.8%
	Usually	95	24.2%
	Always	40	10.2%
	Unsure/don't know	23	5.9%
Move the cat to a more suitable location (e.g. from a centre into a foster home/from one centre to another)	Never	36	9.2%
	Sometimes or occasionally	200	50.9%
	Usually	88	22.4%
	Always	46	11.7%
	Unsure/don't know	23	5.9%
Other	Never	38	10.1%
	Sometimes or occasionally	99	26.2%
	Usually	32	8.5%
	Always	17	4.5%
	Unsure/don't know	192	50.8%

Note: Percentages are based on 393 non-missing responses.

Q55. For cats that have medical issues which are not life threatening but would make them difficult to successfully home with a member of the public, how often might they:

Outcomes	Response	n	Percent

Be homed to a staff member or volunteer	Never	20	6.0%
	Sometimes or occasionally	253	76.2%
	Usually	28	8.4%
	Always	7	2.1%
	Unsure/don't know	24	7.2%
Be kept at your location (i.e. foster room/pen/site/cattery/centre) permanently	Never	218	65.7%
	Sometimes or occasionally	72	21.7%
	Usually	18	5.4%
	Always	4	1.2%
	Unsure/don't know	20	6.0%
Be kept at your location indefinitely, until the right home is found for the cat	Never	60	18.1%
	Sometimes or occasionally	94	28.3%
	Usually	84	25.3%
	Always	79	23.8%
	Unsure/don't know	15	4.5%
Be kept at your location for a limited time, until the right home is found for the cat or their wellbeing deteriorates	Never	33	9.9%
	Sometimes or occasionally	109	32.8%
	Usually	98	29.5%
	Always	66	19.9%
	Unsure/don't know	26	7.8%

Be transferred to a different location (i.e. to a different fosterer/site/cattery/centre) within your organisation or even to a different organisation to improve their chances of finding a suitable home	Never	34	10.2%
	Sometimes or occasionally	195	58.7%
	Usually	60	18.1%
	Always	26	7.8%
	Unsure/don't know	17	5.1%
Be euthanised	Never	192	57.8%
	Sometimes or occasionally	108	32.5%
	Usually	8	2.4%
	Always	0	0.0%
	Unsure/don't know	24	7.2%

Note: Percentages are based on 332 non-missing responses.

Q56. For cats that have behavioural issues which would make them difficult to successfully home with a member of the public, how often might they:

Outcomes	Response	n	Percent
Be homed to a staff member or volunteer	Never	38	11.4%
	Sometimes or occasionally	255	76.8%
	Usually	9	2.7%
	Always	2	0.6%
	Unsure/don't know	28	8.4%
Be kept at your location (i.e. foster room/pen/site/cattery/centre) permanently	Never	218	65.7%

	Sometimes or occasionally	66	19.9%
	Usually	20	6.0%
	Always	6	1.8%
	Unsure/don't know	22	6.6%
Be kept at your location indefinitely, until the right home is found for the cat	Never	62	18.7%
	Sometimes or occasionally	123	37.0%
	Usually	76	22.9%
	Always	48	14.5%
	Unsure/don't know	23	6.9%
Be kept at your location for a limited time, until the right home is found for the cat or their wellbeing deteriorates	Never	31	9.3%
	Sometimes or occasionally	124	37.3%
	Usually	93	28.0%
	Always	51	15.4%
	Unsure/don't know	33	9.9%
Be found an alternative home to a typical domestic environment or 'pet' home	Never	5	1.5%
	Sometimes or occasionally	156	47.0%
	Usually	111	33.4%
	Always	35	10.5%
	Unsure/don't know	25	7.5%
Be transferred to a different location (i.e. to a different fosterer/site/cattery/centre) within your organisation or even to a	Never	40	12.0%

different organisation to improve their chances of finding a suitable home			
	Sometimes or occasionally	209	63.0%
	Usually	46	13.9%
	Always	14	4.2%
	Unsure/don't know	23	6.9%
Be euthanised	Never	176	53.0%
	Sometimes or occasionally	113	34.0%
	Usually	6	1.8%
	Always	0	0.0%
	Unsure/don't know	37	11.1%

Note: Percentages are based on 332 non-missing responses.

Q50. Are any methods used to assess a prospective adopter's suitability to home a particular cat?

Response	n	Percent
Yes	312	94.0%
No	3	0.9%
Unsure/don't know	17	5.1%

Note: Percentages are based on 332 non-missing responses.

Q53. Are any post adoption follow ups undertaken?

Response	n	Percent
Never	4	1.2%
Sometimes or occasionally	46	13.9%
Usually	59	17.8%
Always	211	63.6%

Response	n	Percent
Unsure/don't know	12	3.6%

Note: Percentages are based on 332 non-missing responses.
